# Seasonal nitrogen fluxes of the Lena River Delta

**DOI:** 10.1007/s13280-021-01665-0

**Published:** 2021-12-16

**Authors:** Tina Sanders, Claudia Fiencke, Matthias Fuchs, Charlotte Haugk, Bennet Juhls, Gesine Mollenhauer, Olga Ogneva, Paul Overduin, Juri Palmtag, Vasily Povazhniy, Jens Strauss, Robyn Tuerena, Nadine Zell, Kirstin Dähnke

**Affiliations:** 1 Institute for Carbon Cycles, Helmholtz-Zentrum Hereon, Geesthacht, Germany; 2 Institute of Soil Science, Universität Hamburg, Allende-Platz 2, 20146 Hamburg, Germany; 3 Center for Earth System Research and Sustainability, Universität Hamburg, Allende-Platz 2, 20146 Hamburg, Germany; 4 Permafrost Research Section, Helmholtz Centre for Polar and Marine Research, Alfred Wegener Institute, Telegrafenberg A 45, Potsdam, Germany; 5 Department of Environmental Science and Analytical Chemistry, Stockholm University, Svante Arrhenius Väg 8, 11418 Stockholm, Sweden; 6 Marine Geochemistry Section, Helmholtz Centre for Polar and Marine Research, Alfred Wegener Institute, Am Handelshafen 12, 27570 Bremerhaven, Germany; 7 Department of Geography and Environmental Sciences, Northumbria University, Newcastle-upon-Tyne, NE1 8ST UK; 8 Otto Schmidt Laboratory for Polar and Marine Research, Arctic and Antarctic Research Institute, Beringa 38, Saint Petersburg, Russia 199397; 9 Scottish Association for Marine Science, Dunstaffnage, Oban PA37 1QA UK

**Keywords:** Arctic Ocean, Lena Delta, Nitrogen, Nitrous oxide

## Abstract

The Arctic is nutrient limited, particularly by nitrogen, and is impacted by anthropogenic global warming which occurs approximately twice as fast compared to the global average. Arctic warming intensifies thawing of permafrost-affected soils releasing their large organic nitrogen reservoir. This organic nitrogen reaches hydrological systems, is remineralized to reactive inorganic nitrogen, and is transported to the Arctic Ocean via large rivers. We estimate the load of nitrogen supplied from terrestrial sources into the Arctic Ocean by sampling in the Lena River and its Delta. We took water samples along one of the major deltaic channels in winter and summer in 2019 and sampling station in the central delta over a one-year cycle. Additionally, we investigate the potential release of reactive nitrogen, including nitrous oxide from soils in the Delta. We found that the Lena transported nitrogen as dissolved organic nitrogen to the coastal Arctic Ocean and that eroded soils are sources of reactive inorganic nitrogen such as ammonium and nitrate. The Lena and the Deltaic region apparently are considerable sources of nitrogen to nearshore coastal zone. The potential higher availability of inorganic nitrogen might be a source to enhance nitrous oxide emissions from terrestrial and aquatic sources to the atmosphere.

**Supplementary Information:**

The online version contains supplementary material available at 10.1007/s13280-021-01665-0.

## Introduction

The Arctic is warming at twice the rate of the global average (Smith et al. 2019). In the Arctic Ocean, for instance, sea ice, especially multi-year ice, is being lost at an unprecedented rate (Overland and Wang 2013). Longer ice-free periods result in potentially higher productivity and changes of food webs (Lewis et al. 2020). At the same time, increasing air temperatures are thawing permafrost around the Arctic Ocean (Biskaborn et al. 2019), which then release organic matter including nitrogen to river and further on to the Arctic Ocean.

The permafrost-affected soils store a globally large amount of organic matter, including organic carbon (OC) and nitrogen (N). It is estimated that more than half of the global soil organic matter (SOM) are stored within the northern permafrost region. Hugelius et al. (2014) estimated a total SOC stocks in the northern circumpolar permafrost region of ∼1300 Pg ± 200 Pg. A recent publication by Mishra et al. (2021) based on > 2700 soil profiles estimated a total SOC stock of 1000 (− 170 to + 186 Pg) to 300 cm. Circumpolar estimate for N ranged between 40 and 67 Pg N (Weintraub and Schimel 2003; Harden et al. 2012). Compared to SOC stocks, we know little about the N stocks in the permafrost zone, which are therefore a subject of ongoing debate. For instance, based on typical soil C:N ratios of 10–50 and scaled to the amount of SOC, the actual N stock in permafrost-affected soils could reach 130 Pg N (Voigt et al. 2020). Abbott and Jones (2015) suggest that globally relevant quantities of N are stored in permafrost soils.

These stocks are prone to be reactivated by thawing ground ice, which leads to a suite of mass wasting and subsidence processes such as thermokarst. The release of N stocks also results from fluvial (Kanevskiy et al. 2016; Fuchs et al. 2020) and coastal erosion (Günther et al. 2013). In order to understand N release and its utilization as a consequence of permafrost thaw, we need studies that combine investigations of soil N cycling with soil erosion, soil release of reactive nitrogen to the rivers, and transport to the Arctic Ocean. Reactive nitrogen are components (dissolved inorganic N (DIN) and dissolved organic N (DON)), which are available for primary producers such as plants and microorganisms.

There are some studies on N cycling in permafrost-affected soils (e.g., Biasi et al. 2005; Stewart et al. 2014; Sanders et al. 2019; Horn and Hetz 2021). Recent newer studies focus on the potential nitrous oxide (N_2_O) emissions from permafrost-affected soils (reviewed by Voigt et al. (2020)) and the input of C and N into the fluvial system (Hugelius et al. 2020; Pastor et al. 2020). While most N turnover occurs during summer, winter N mineralization may provide an important N source upon thaw in spring, when a pulse of nitrate (NO_3_^−^) in soil solution has been observed in studies of Arctic ecosystems (Schimel and Bennett 2004; Buckeridge et al. 2013; Rasmussen et al. 2020). Inorganic dissolved nitrogen or small dissolved organic N compounds such as urea or amino acids can contribute to primary productivity in soils (Schimel and Bennett 2004) and nevertheless, nitrogen and phosphorus are limiting nutrients in the Arctic ecosystems such as soils and rivers (Shaver et al. 1986; Beermann et al. 2014; Schade et al. 2016; Hobbie et al. 2021).

Other studies have investigated N fluxes in Arctic rivers (Dittmar and Kattner 2003; Frey and McClelland 2009; Holmes et al. 2012); rivers receive a large portion of N from soils during the spring freshet, which is consumed during summer (Buckeridge et al. 2010; Abbott and Jones 2015). N is mainly transported to the Arctic Ocean as DON and NO_3_^−^ (Holmes et al. 2012). The mean riverine NO_3_^−^ contribution to ocean primary production in the Arctic is generally low and is approx. 5% for the Laptev Sea (Le Fouest et al. 2015). Both nitrite (NO_2_^−^) and ammonium (NH_4_^+^) are rapidly converted into NO_3_^−^ or assimilated by plants or phytoplankton. However, rapid uptake of DIN, coupled with relatively quick regeneration of dissolved organic nitrogen (DON) in N‐limited nearshore regions, could potentially lead to high local rates of riverine‐supported photosynthesis (Tank et al. 2012). Arctic rivers annually carry ~ 13% of the freshwater transported globally from land to ocean, though the Arctic Ocean only makes up approximately 1% of the total global ocean volume. (Aagaard and Carmack 1989; Anderson et al. 1998). Throughout the Eurasian Shelf region, rapid summertime depletion of NO_3_^−^ across the full fluvial–marine transition in the Laptev Sea indicates N limitation (Kattner et al. 1999; Dittmar and Kattner 2003; Reyes and Lougheed 2015).

Across the entire Arctic, the influx of DON to the Arctic shelf waters from rivers is five times greater compared to the influx of NO_3_^−^ (Dittmar and Kattner 2003) and also 70% of the DON is removed in shelf waters before even reaching the open marine environment (Thibodeau et al. 2017). In the nearshore regions, the riverine nutrients (organic and inorganic) have the greatest influence on productivity (Dunton et al. 2006). However, Tank et al. (2012) found that riverine nutrients are not sufficient to explain a large proportion of primary production in the Arctic Ocean. Although, Terhaar et al (2021) reported recently that riverine inputs indeed fuels primary production, but that satellite-based primary production rates might be overestimated especially for the Laptev and East Siberian Seas. It is important, therefore, to understand how much permafrost degradation may impact N forms and amounts from delta to nearshore environments, and the potential changes to the riverine and coastal nitrogen cycle and the ratio between its organic and inorganic N components. Generally, estuarine and deltaic regions are filters for riverine inputs to the coastal waters and the open ocean (Smith et al. 2005). They can also be a considerable source of N_2_O emissions (Seitzinger and Kroeze 1998; Dunton et al. 2006; Marzadri et al. 2021), nevertheless, N_2_O emissions from Arctic rivers and deltas are as yet poorly investigated.

In this study, we want to assess the amount of nitrogen which is released and transported from terrestrial sources to the Lena River and further through its delta towards the nearshore Laptev Sea. Based on our findings, we discuss the implications that thawing permafrost and release of reactive nitrogen may have on the ecosystem and the nitrogen cycle. In addition, we explore whether the increasing availability of reactive inorganic nitrogen in soils and rivers can potentially cause increasing N_2_O emissions.

## Materials and methods

In this study, we present nutrient data focusing on N from two cruises in winter and summer 2019 from the inner Lena Delta to the nearshore Laptev Sea and a one-year monitoring data set from the Research Station Samoylov in 2018 and 2019 (see (Juhls et al. 2020)). Furthermore, in order to estimate soil release of organic matter including C and N, soil incubation experiments were conducted. We measured the release of inorganic N forms by remineralization and nitrification and potential nitrous oxide (N_2_O) production.

### River study site and cruises

**Fig. 1:**
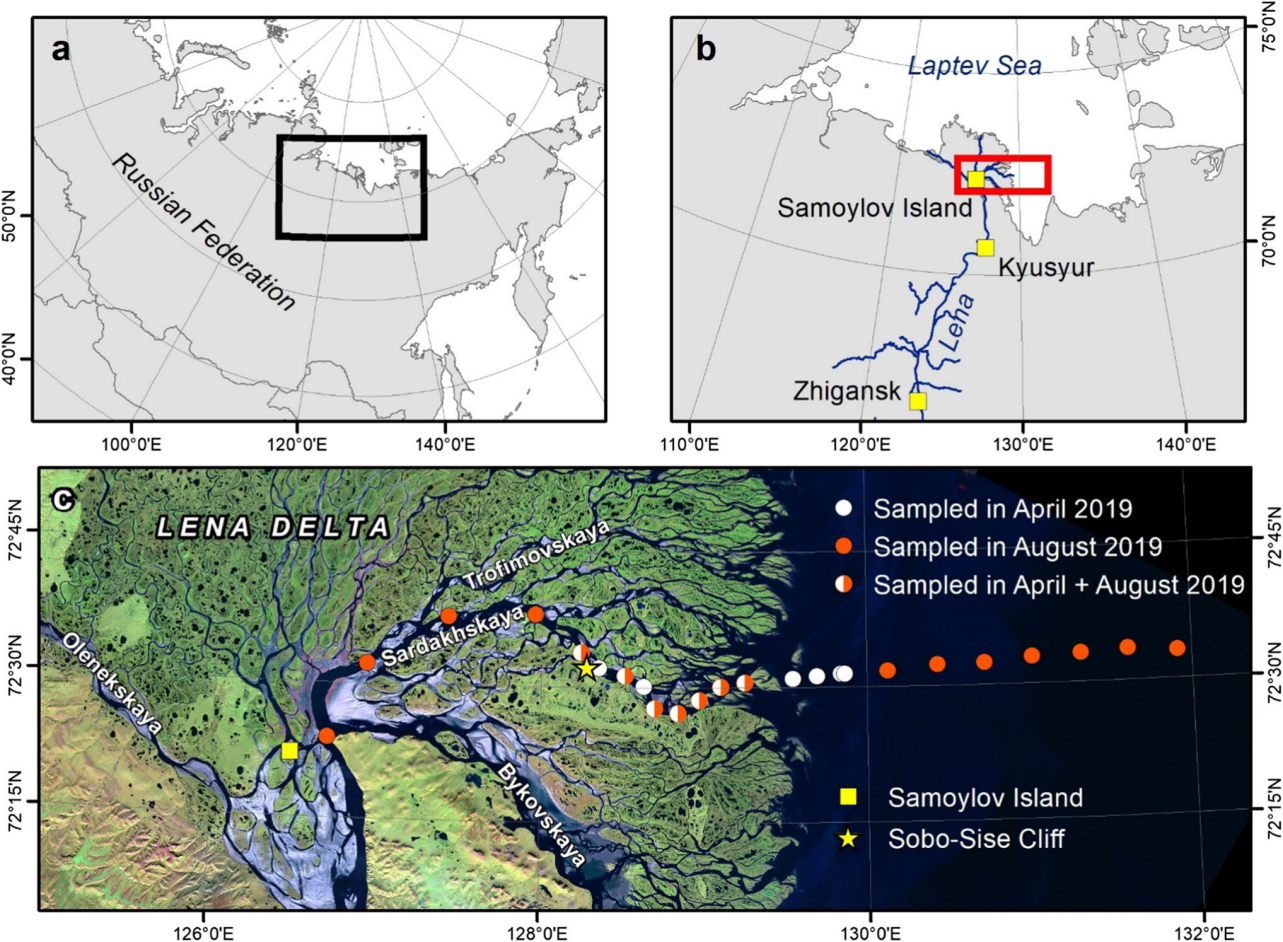
Study site area of the Lena Delta in Northeast Siberia (**a**). Lena River and Delta including the ArcticGRO station in Zhigansk, the gauge station in Kyusyur, and the Samoylov Station (**b**). Transect and sampling sites of the cruises in winter (white dots) and summer (red dots), Samoylov Island, sampling site for soil incubations and the one-year data set, Sobe-Sibe Cliff (**c**)

The Lena Delta is located in northeastern Siberia, where the Lena River cuts through the Verkhoyansk Mountains range and discharges into the Laptev Sea, a shallow shelf sea in the Arctic Ocean. The Lena is divided in three major channels, the Olenekskaya flowing to the western Laptev Sea and the Trofimovskaya, which bifurcates into the Sardakhskaya and Bykovskaya channels, both flowing into the eastern Laptev Sea (Fig. 1) (Fedorova et al. 2015). Samples were collected along the Sardakhskaya Channel in late winter (March/April 2019) and summer (August 2019). The Sardakhskaya Channel has a water discharge of ~ 8000 m^3^ s^−1^, which represent up to 40% of the discharge of the Lena main channel (Fedorova et al. 2015). The Lena River is ice-covered for about 8 months a year between October and May with an ice thickness of up to 2 m. Water depth at the beginning of the Sardakhskaya Channel can reach 22 m (Fedorova et al. 2015) and is approximately 11 m in front of Sobo-Sise cliff (Fuchs et al. 2020), so that water flows underneath the river ice cover during the winter months (Fuchs et al. (in review)).

Water, suspended particulate matter (SPM), and surface sediment samples were collected. Overall, 24 stations were sampled: at seven sites were sampled in both winter and summer, with an extra six sites sampled in winter only, and an extra 11 sites sampled in summer only (Fig. 1C).

In winter, water samples were collected from beneath the ice, if possible, additional on the halfway down and above the sediment through holes drilled through the river ice (ca. 2 m thickness) using an UWITEC water sampler. Holes were drilled at 5-km intervals until approximately 30 km offshore. Conductivity, temperature, and depth (CTD) were measured at each station using a SontekTM CastAway CTD sensor. Water samples were filtered directly in the field through pre-combusted GF/F glass fiber filters (0.7 µm, Whatman) and stored frozen in HDPE bottles until thawed for further analysis.

In summer, the transect was sampled in two short campaigns: (1) from offshore to the nearshore in the Laptev Sea with the marine vessel *Anatoliy Zhilinskiy* and (2) from the board of the river ship *Merzlotoved* along the Sardakhskaya channel. In the Laptev Sea, we sampled the water column at three different depths, 1 m below the water surface, halfway down the water column, and just above the riverbed or seabed. In the Lena Delta, one to three water samples were taken depending on the water depth. Additionally, sediment samples were taken using a gravity corer (from which the overlying bottom water was sampled).

121 river water samples were collected at the Samoylov research station from September 2018 to September 2019. The sampling procedure is described in detail in Juhls et al. (2020). Water samples were collected from just below the river surface in the center of the Olenekskaya Channel near Samoylov Island in a pre-rinsed HDPE bottle. Samples were collected from a boot during the ice-free period and from the ice during the winter. When ice break-up or freeze-up made access dangerous, samples were taken from the shore of Samoylov Island.

### Soil study site and incubations

**Fig. 2:**
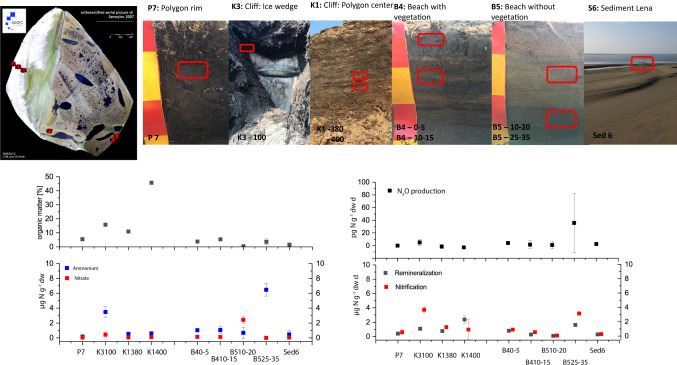
Properties of investigated soils and their remineralization, nitrification and N_2_O production rates. Soil samples were taken at 6 different sites: K1 (cliff, former polygon center) in two depth 3.8 and 4.0 m below the ground surface, K2 (cliff, former polygon wall, close to the ice wedge), 1.0 m below the ground surface, P7 (polygon wall), depth 10–20 cm, B4 (beach, primary vegetation cover), 0–5 cm and 10–20 cm, B5 (beach without vegetation cover), 10–20 cm and 25–35 cm, Sed6 (sediment in the Lena) as surface sediment. Organic matters were measured by combustion at 550 °C; NO_3_^−^ and NH_4_^+^ were extracted by 0,0125 M KCl; remineralization and nitrification rates were measured by incubation of soil in Lena water; N_2_O production rates were measured after soil samples were transported to Germany

Soil was sampled from different soil types and geomorphologic units on Samoylov Island. The soils of Samoylov represent the main Holocene soil types in the younger part of the Lena Delta. The island can be divided into two major geomorphological units (Sanders et al. 2010), which vary in moisture regime and in SOM (Boike et al. 2013) (Fig. 2). The western part of Samoylov Island represents a relatively young floodplain up to 4 m above river level (a.r.l.), which is flooded in spring. The eastern part of Samoylov Island is an elevated (10–16 m a.r.l.) river terrace of late Holocene age. The river terrace is flooded only during extreme flooding events (Kutzbach et al. 2004). Soil types and characteristics are described in detail elsewhere (Sanders et al. 2010; Zubrzycki et al. 2013).

Soils were sampled from the floodplain and elevated river terrace. For the measurement of the OM content, extractable DIN, and the incubation setups, soil was sampled from polygonal tundra, cliff, and beach at seven sites (Fig. 2). Permafrost was sampled from the cliff face of Samoylov Island on the river terrace (K1 and K3). The cliff was exposed by ongoing thawing and erosion. At K1, soil was sampled from a former polygon center in a depth of 3.8 and 4 m below the ground surface. In addition, the polygonal tundra (P7) were sampled at a polygon rim in 10–20 cm. At K3 soils were sampled next to an ice wedge and originated from a former polygon rim in 1 m below the ground surface. Furthermore, a transect was sampled from the floodplain to the beach into the Lena River sediment at different depths (B4: 0–5 cm and 10–15 cm, B5: 10–20 cm and 25–35 cm, and S6: surface sediment). Primary vegetation was present only at sampling site B4. The other sites were bare soil (Fig. 2).

Soil samples were incubated in Lena water to quantify the remineralization and nitrification of organic N to inorganic N (NH_4_^+^ and NO_3_^−^). We followed two different approaches for sample analysis: (1) Analyses during the field campaign in 2019 in the laboratory of the Samoylov Island Research Station to measure the net N-remineralization and net nitrification rates; (2) Analyses of frozen soil samples and Lena River water at the University of Hamburg to measure potential N_2_O production.

For the first analysis, 15–20 g of wet soils and 130 ml unfiltered Lena water were incubated on a shaker (14 days, 110 rpm) at room temperature (20–25 °C). Subsamples of mixed overlying water were taken every 2–3 days. At the end of the incubation, the remaining soil/sediment and the overlying water were sampled. To calculate net N remineralization and net nitrification, DIN was measured over time, and sediment and soil TN were determined at the end of the incubations.

In another incubation, we measured potential aerobic nitrous oxide production rates. 1 g fresh weight of homogenized soil was weighed in 100 ml serum bottles. 20 ml Lena River water was added and the bottles were sealed air-tight with rubber septa. Soil samples were incubated in 18 replicates at 5 °C, without shaking in the dark. 3 × 1 ml gas samples were taken after 1, 2, 8, 11, and 18 weeks, and N_2_O concentration was determined by gas chromatography (GC, Agilent Technologies 7890 A, Santa Clara, CA, USA).

### Laboratory analyses

For this study, water, suspended particulate matter (SPM), and sediment were sampled. Soil samples were taken for incubation experiments. Dissolved nutrients (NH_4_^+^, NO_2_^−^, NO_3_^−^), phosphate and silicate, total dissolved organic nitrogen and phosphorus (TDN and TDP), and dissolved carbon (DOC) were measured in filtered water samples. Total nitrogen and phosphorus (TN and TP) were measured in unfiltered water samples. SPM was analyzed for C and N content and δ^15^N. Sediment samples were analyzed for grain size, C and N content and δ^15^N. Soil and sediment samples from Samoylov Island were analyzed for their OM, C, N, δ^15^N and extractable dissolved inorganic N contents. A detailed description of the analytical methods is provided in the Supplementary Information.

### Estimation of nitrogen loads from the Lena River to the nearshore Laptev Sea

We used the Samoylov monitoring data of 121 samples from September 2018 to August 2019 to calculate the annual N-NO_3_^−^ and TN flux to the Arctic Ocean. We linearly interpolated between sampling days to obtain daily concentrations. To calculate discharge, we corrected the Lena River discharge data from the Roshydromet gauge station at Kyusyur (Shiklomanov et al. 2021.) for the travel time to the sampling station Samoylov Island (Juhls et al. 2020) Fig. 6). Daily concentration was multiplied with corrected daily discharge values and summed to calculate annual fluxes.

## Results

### Hydrographic properties from land to ocean

**Fig. 3:**
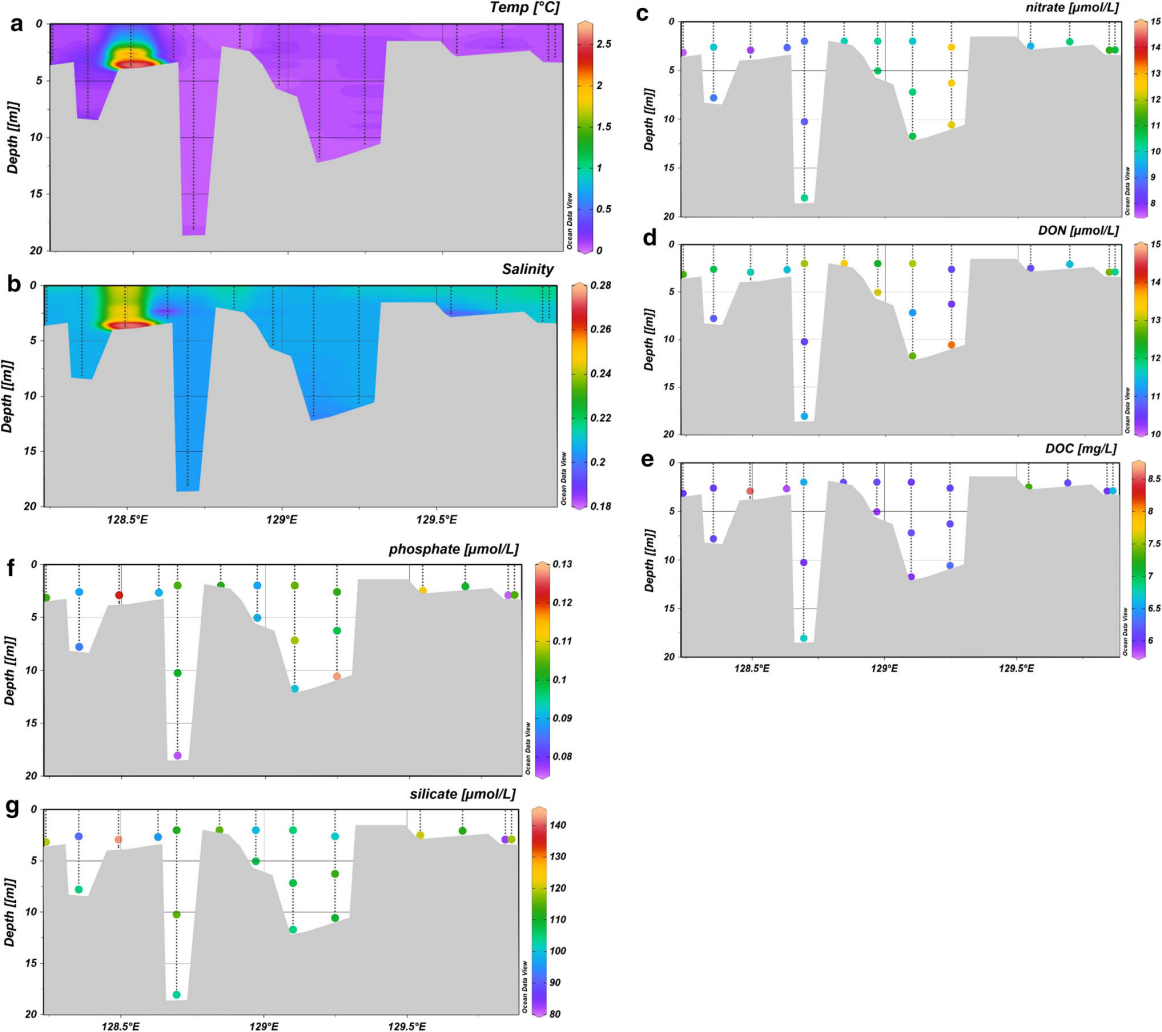
Water properties and nutrient concentrations from the winter cruise. (**a**) temperature (°C), (**b**) salinity (PSU), (**c**) nitrate (µmol/L), (**d**) dissolved organic nitrogen (DON) (µmol/L), (**e**) dissolved organic carbon (DOC) (mg/), (**f**) phosphate (µmol/L), and (**g**) silicate (µmol/L)

During the winter cruises, we investigated a transect just in the Sardakhskaya channel. Water depth varied between 2 and 18 m. Water temperature and salinity profiles showed little variation, with range from 0.05 to 0.18 °C and 0.19 to 0.21 PSU (excluding site CAC19-F) (Fig. 3a + b). Measurements from site CAC19-F were anomalously warm (2.17 °C) with higher salinity values (0.25 PSU) relative to all other samples.

**Fig. 4:**
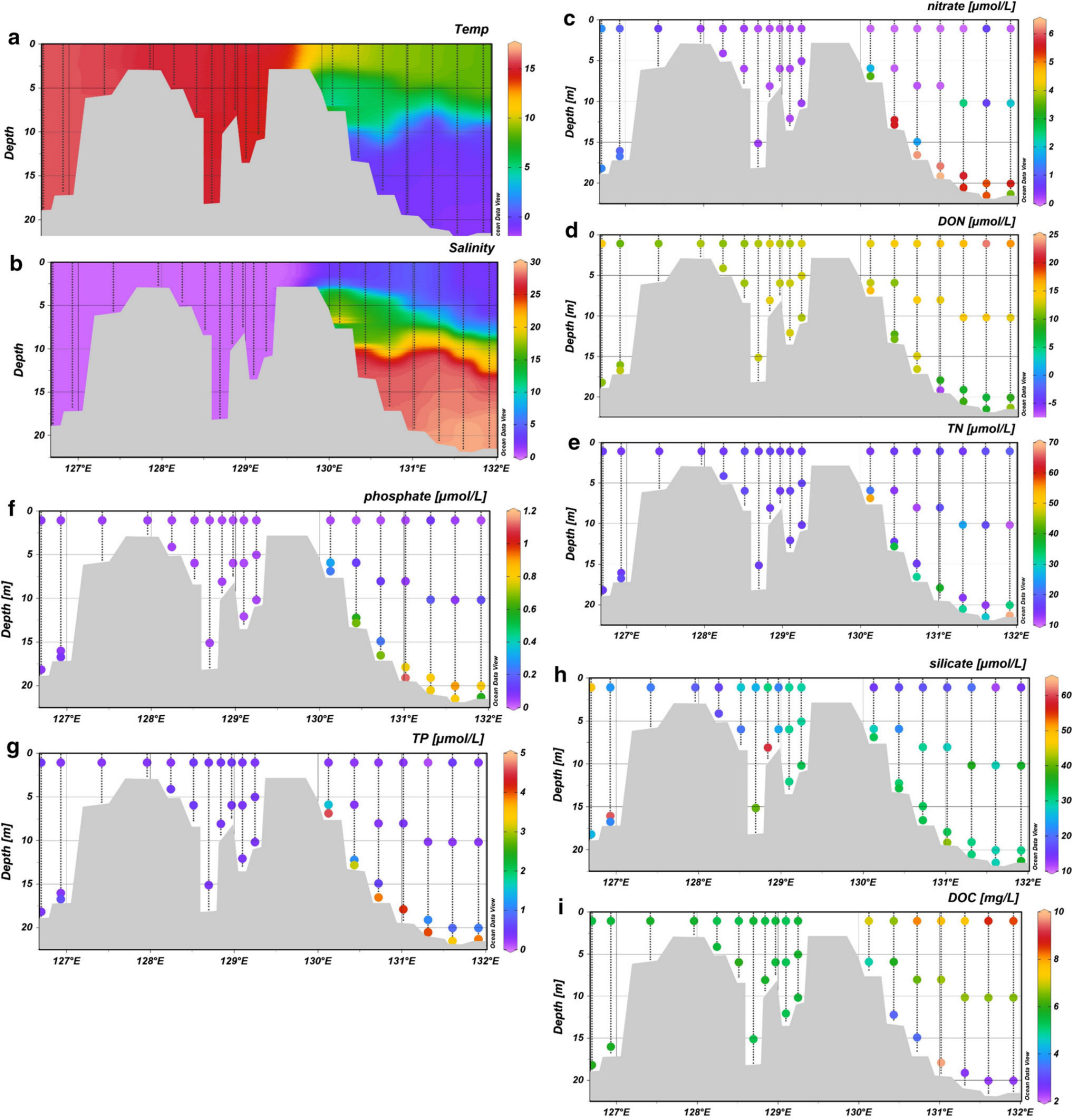
Water properties and nutrient concentrations from the summer cruise. (**a**) Temperature (°C), (**b**) salinity (PSU), (**c**) nitrate (µmol/L), (**d**) dissolved organic nitrogen (DON) (µmol/L), (**e**) total nitrogen (TN) (µmol/L), (**f**) phosphate (µmol/L), (**g**) total phosphate (TP) (µmol/L), (**h**) silicate (µmol/L), and (**i**) dissolved organic carbon (DOC) (mg/)

During the summer cruises, we investigated a longer transect from the center of the delta to approx. 80 km outside the delta in the nearshore Laptev Sea. The water depth ranged from 20 m in the main channel, to less than 0.5 m at the edge of the delta. In the coastal Laptev Sea, water depths gradually increased to 22 m. The temperature and salinity were homogenous in the water column within the delta compared to a strongly stratified water column in the coastal Laptev Sea. Salinity ranged from 0.1 in the delta to 30 in the deeper Arctic shelf water. Temperature ranged from 16 °C in the delta to − 1.0 °C in the bottom water. (Fig. 4a + b).

The data of the winter and summer cruises are accessible in the Pangaea data base (https://doi.pangaea.de/10.1594/PANGAEA.933187) (Fuchs et al. (in review)).

### Sediment properties from land to ocean

The riverbed of the main channels near Samoylov and in the Sardakhskaya Channel consists of mostly sandy sediments with a sand content of over 98%. The sediments in the nearshore Laptev Sea are muddier with a sand content below 10% and a silt content of over 50%. The organic matter content of sediments in the delta is very low (< 0.2%). In the Laptev Sea, OC was between 2 and 3% and δ^15^N values were slightly enriched in relation to the soils within the delta and ranged from 3.0 to 3.7‰ (Table 1).

**Table 1:** Sediment properties during the summer cruises in August 2019

Sample ID	Longitude	Latitude	Sand (%)	Silt (%)	Clay (%)	Total nitrogen (%)	Total nitrogen (µg/g)	Total carbon (%)	Total carbon (µg/g)	δ^15^N (‰)	C/N
Lena delta											
CAC19-Lena 1	126.69564	72.39938	99.7	0.2	0.1	0.01	0.06	0.1	1.1	n.d	18.7
CAC19-Lena 2	126.92899	72.53734	99.6	0.3	0.1	0.01	0.08	0.2	2.2	n.d	27.1
CAC19-Lena 3	127.41932	72.62705	99.6	0.3	0.1	0.01	0.06	0.1	0.8	n.d	13.8
CAC19-Lena 4	127.95918	72.63351	99.5	0.4	0.0	0.01	0.06	0.1	1.1	n.d	18.5
CAC19-Lena 5	128.24466	72.56380	n.d	n.d	n.d	n.d	n.d	n.d	n.d	n.d	n.d
CAC19-Lena 6	128.51548	72.52111	n.d	n.d	n.d	n.d	n.d	n.d	n.d	n.d	n.d
CAC19-Lena 7	128.69504	72.46133	97.8	1.0	1.2	0.01	0.07	0.1	0.8	n.d	11.3
CAC19-Lena 7/8	128.84105	72.45303	99.0	0.4	0.6	0.00	0.04	0.1	1.1	n.d	27.3
CAC19-Lena 8	128.97076	72.47704	98.4	0.8	0.8	0.01	0.07	0.1	1.1	n.d	16.1
CAC19-Lena 8/9	129.09922	72.50168	n.d	n.d	n.d	n.d	n.d	n.d	n.d	n.d	n.d
CAC19-Lena 9	129.24841	72.50904	98.3	0.8	0.9	0.01	0.06	0.1	0.6	n.d	10.0
Laptev sea											
CAC19-S-04	130.12630	72.53012	1.7	83.2	15.0	0.1	1.4	2.3	22.5	3.0	15.7
CAC19-S-05	130.43350	72.53983	10.1	67.5	22.4	0.2	1.6	2.3	23.4	3.3	14.4
CAC19-S-06	130.72248	72.54118	2.9	68.7	28.4	0.2	1.6	2.3	22.9	3.1	14.6
CAC19-S-07	131.01836	72.55056	2.3	66.1	31.6	0.2	1.6	2.2	22.1	3.2	13.9
CAC19-S-08	131.31468	72.55446	5.1	55.4	39.5	0.2	2.0	2.7	26.5	3.6	13.1
CAC19-S-09	131.60606	72.55886	0.6	56.7	42.8	0.2	2.2	2.8	28.0	3.7	12.8
CAC19-S-10	131.91480	72.55320	2.7	58.6	38.7	0.2	1.8	2.2	22.0	3.6	12.0

*n.d.* not determined. *CN* ratio of TN and TC

### Nutrients from land to ocean

Nutrient concentrations differed considerably between the winter and summer sampling campaigns. In winter, NO_3_^−^ and DON were present along the transect. In summer, NO_3_^−^ was near the detection limit, DON and particulate N are dominant forms of N in the study region.

In winter, NO_3_^−^ concentrations increased from the delta interior to the nearshore by ~ 3 µmol L^−1^ from 7.5 to 10.5 µmol L^−1^. DON remained relatively constant through the transect (10 ± 1 µmol L^−1^), contributing approximately 50% of the dissolved N pool. NH_4_^+^concentrations were below 1.5 µmol L^−1^ in all samples apart from CAC19-F (14.6 µmol L^−1^), suggesting that this sample may have been influenced by riverbed sediments.

DOC concentrations were generally constant along the river-to-sea transect for all winter samples (except CAC19-F), with a mean value of 6.2 mg L^−1^ and a minimum of 5.8 mg L^−1^ (Fig. 3).

In summer, the NO_3_^−^ concentration was considerably lower than in winter. There was a decrease in NO_3_^−^ (1.4 to 0.2 µmol L^−1^) from the delta interior and in the beginning of the Sardakhskaya channel and a slight increase in DON (10 to 13 µmol L^−1^). The bottom water in the Laptev Sea had a NO_3_^−^ concentration of approx. 5 µmol L^−1^ (Fig. 4).

### Nutrients at the Samoylov monitoring station

**Fig. 5:**
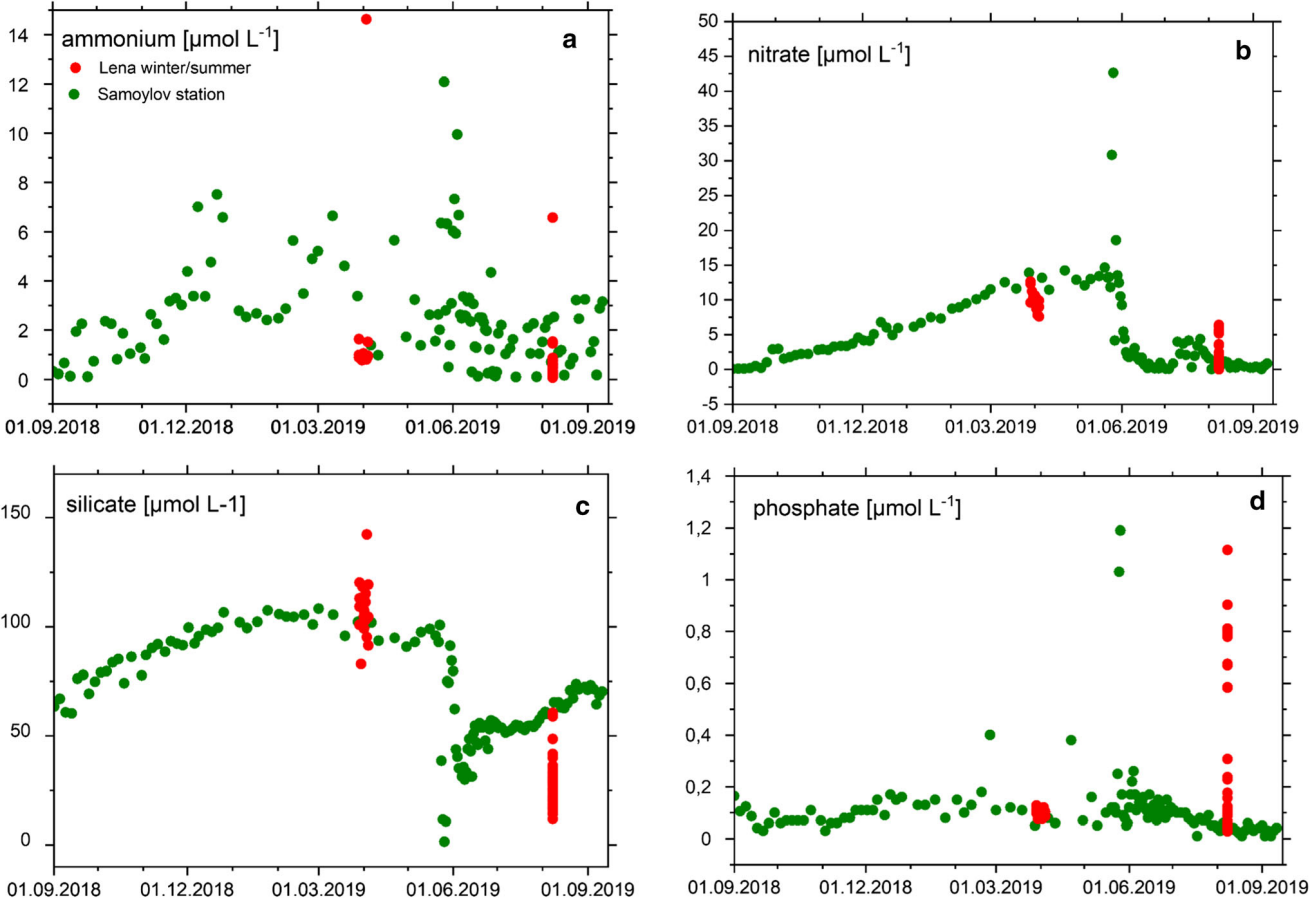
Nutrient data from Samoylov Monitoring station September 2018–September 2019 (green) and data from the two Lena cruises in winter and summer 2019 in this study (red). **a** Ammonium (µmol/L), **b** nitrate (µmol/L), **c** silicate (µmol/L), and **d** phosphate (µmol/L)

**Fig. 6:**
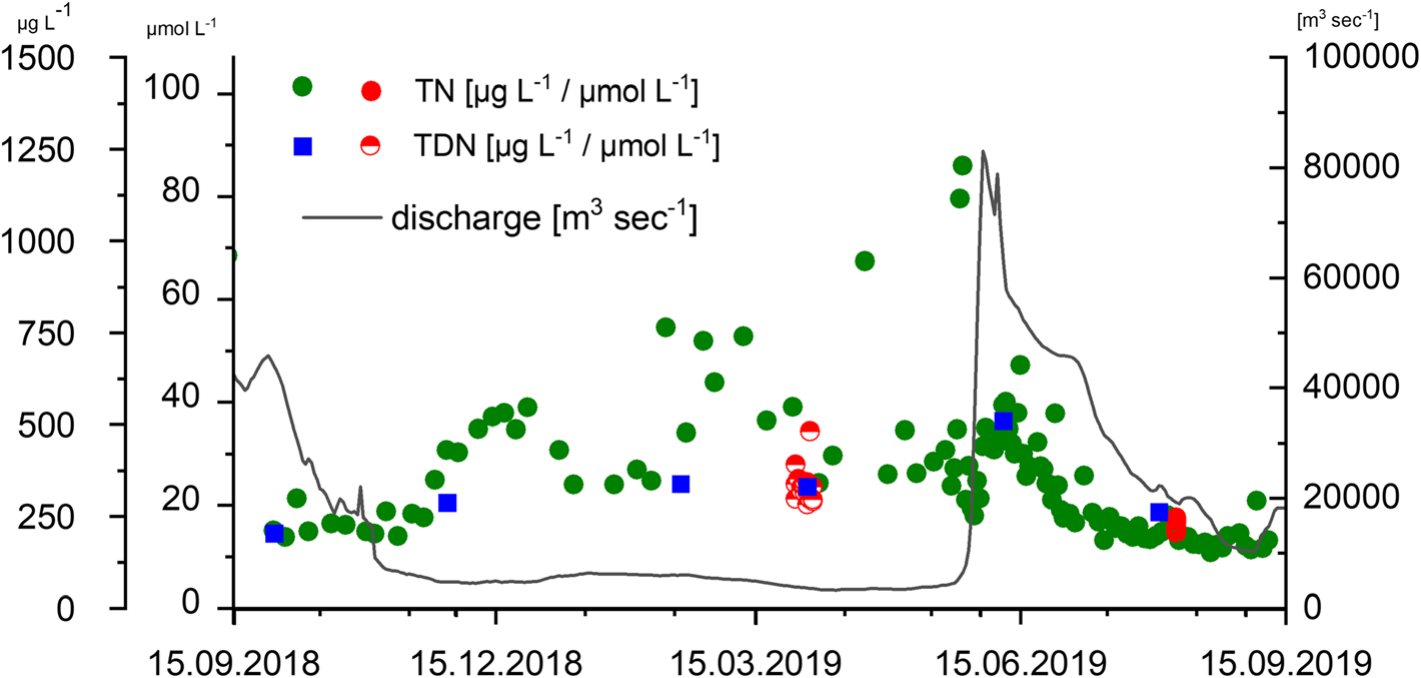
Total N (µg L^−1^ and µmol L^−1^) concentration at the Samoylov Monitoring (green) from 15.9.2018 to 15.9.2019 and from the summer cruises (red, sites inside the delta). TDN (µg L^−1^ and µmol L^−1^) concentration from the winter cruises (half-red circles) and from the ArcticGRO station (blue square, Holmes et al. 2021) from 15.9.2018 to 15.9.2019. Discharge (black line) in m^3^ sec^−1^ at the gauge station Kyusyur

At the Samoylov monitoring station, nutrient concentrations were measured from September 2018 to September 2019, including nitrate + nitrite (herein presented as NO_3_^−^), NH_4_^+^, phosphate (PO_4_^**3−**^), silicate, and TN (Figs. 5, 6). NO_3_^−^ was detectable during periods of ice cover from October to June, reached highest concentration during the spring flood and then dropped below the detection limit. The NH_4_^+^ concentrations ranged from below detection limit up to 12 µmol L^−1^. There was a clear increase in NH_4_^+^ when the Lena ice cover built up from October to December. The phosphate^−^ concentrations ranged from below the detection limit up to 0.4 µmol L^−1^, during the spring flood the concentration increased to more than 1.0 µmol L^−1^. The silicate concentration showed a clear seasonal cycle with an increase during the ice-covered months from approx. 50 µmol L^−1^ to slightly above 100 µmol L^−1^. The TN (DIN + DON + particulate N) values (Fig. 6) showed a similar pattern with higher but more variable concentrations in winter and lower concentration in summer. From September 2018 to August 2019, we estimated an annual N-NO_3_^−^ load of 24.5 Gg year^−1^ and a total N load of 242.1 Gg year^−1^.

### Nutrient and potential N_2_O release and transport from soils to river

We incubated two types of soil samples: firstly, organic-rich and peaty soils of the polygonal tundra which can enter the Lena River after thawing and erosion at the cliffs of the Samoylov Island, and secondly, soils and sediment from the floodplain, which are regularly flooded by the Lena River and contain mostly allochthonous organic matter from the river.

The soil samples differed mainly in SOM, which ranged from 0.5 to 45.7%. SOM of the polygonal tundra and the cliff was generally higher (above 5%) than in soils from the floodplain (0.5–5.8%) (Table 2).

**Table 2:** Soil properties of samples from Samoylov Island

Sample ID	Depth (cm)	Sample site	Sand (%)	Silt (%)	Clay (%)	SOM (%)	Total Nitrogen (%)	Total Nitrogen (µg/g)	Total Carbon (%)	Total Carbon (µg/g)	δ^15^N (‰)	C/N
Polygonal tundra												
LD19P7	10–20	P7	55.9	37.2	6.9	6.0	0.1	1.4	2.5	25.4	0.7	18.4
LD19K3	100	K3	31.9	56.2	11.9	15.8	0.4	3.9	8.9	88.8	0.6	22.8
LD19K1	380	K1	15.7	69.9	14.4	10.9	0.3	2.7	5.5	54.9	1.0	20.3
LD19K2	400	K1	n.d	n.d	n.d	45.7	0.4	3.9	16.2	162.1	0.8	41.3
Floodplain												
LD19B4 0–5	0–5	B4 0–5	49.9	43.1	7.1	3.5	0.1	0.9	1.4	13.7	1.6	15.7
LD19B4 10–15	10–15	B4 10–15	51.5	40.6	7.9	5.8	0.1	1.0	1.6	16.2	1.4	15.9
LD19B5 10–20	10–20	B5 10–20	97.6	1.7	0.7	0.5	0.0	0.1	0.1	1.10	n.d	7.3
LD19B5	25–35	B5 25–35	74.2	21.0	4.7	5.2	0.1	1.0	2.0	19.6	0.9	19.2
LD19Sed6	0–5	Sed 6	91.6	7.4	1.0	2.0	0.0	0.2	0.4	3.8	n.d	16.3

*n.d.* not determined. *CN* ratio of TN and TC

The NH_4_^+^ content of the soils ranged between 0.1 and 6.5 µg N g^−1^ dw. The highest concentration was found in the peat containing cliff and the organic-rich layer of the beach. A considerable amount of NO_3_^−^was detected in the sandier layer of the beach.

The soil and sediment samples from the floodplain were sandy to silty with a sand content between 50 and 98%. Silt content increased with distance from the water line of the river. The soils from the river terrace contained less sand, whereas SOM was higher than in the floodplain samples. The δ^15^N values of river terrace soils were lower than those found in the floodplain (Table 2) and ranged between 0.6 and 1.6 ‰, which is close to the average of the atmospheric N_2_ of 0‰ (Kendall 1998).

In all soil incubations, DIN production occurred. The samples with high NH_4_^+^ concentrations such as K3 near the ice wedge and the deeper sample from the beach (B5 25–35) also had higher NH_4_^+^ concentrations during the incubation and showed an increase of NO_3_^−^ after a maximum of five days. The net N-remineralization rates ranged between 0.1 and 1.7 µg N g^−1^ d^−1^ and they correlated with the organic matter content (*R*^2^ of 0.72) and OC (*R*^2^ of 0.65) (Fig. 2).

The net nitrification rates ranged between 0.1 and 5.0 µg N g^−1^ day^−1^ (Fig. 2). The highest rates were observed in the organic- and ammonium-rich cliff and beach soils. The nitrification rates correlated (*R*^2^ of 0.92) with the NH_4_^+^ concentration.

We also investigated the potential N_2_O production of soils from the delta region. A considerable N_2_O production was only detected in the incubations after 8 weeks at 5 °C in the non-vegetated subsoil samples of the floodplain, containing higher SOM than the topsoil, with mean N_2_O production rate of 35.7 ± 46.5 pg N-N_2_O g dw^−1^ day^−1^ (Fig. 2). In all the other incubations, the N_2_O formation was near the detection limit.

## Discussion

### Nitrogen signal in the Lena Delta

Dissolved N was exported mostly as DON and NO_3_^−^ during the winter, whereas DON prevailed in the delta in summer. These results corroborate observations from the Arctic GRO data from Zhigansk in the south (Holmes et al. 2012, 2021).

In winter (October to May), DON, NH_4_^+^, and NO_3_^−^ were continuously released to the aquatic environment, resulting in relatively high and uniform concentrations of NO_3_^−^ and DON in the water column (Fig. 3C, D and 5). After the growing season, when the active layer freezes, we observed a clear increase of NO_3_^−^ and NH_4_^+^ at the Samoylov station. This allochthonous, terrestrial DIN was not consumed by plants, but rather leached to the river.

With warming and active layer deepening into mineral horizons with low C/N ratios, the hydrological flow path penetrates deeper, intensifying NO_3_^−^ and NH_4_^+^ leaching to the river (Harms and Jones 2012). In soils with vegetation cover, plants and microbial communities will compete for remineralized DON and DIN (Stark and Kytöviita 2006). By the end of the growing season or in unvegetated soils, mineralization and nitrification will be the dominant processes. Therefore, winter N export from soils to rivers in the unfrozen active layer will be in the form of NH_4_^+^, NO_3_^−^, and DON. Especially, because by the end of the vegetation period, NO_3_ was enriched in soils without vegetation cover (Sanders et al. 2010).

In summer, the main N export from soils takes place via DON and NH_4_^+^, which is consistent with former studies in permafrost-affected soils (Harms and Jones 2012). In the river, NH_4_^+^ and NO_3_^−^ are consumed immediately (Dittmar and Kattner 2003), again either by primary producers or oxidized to nitrate. This was confirmed by our own results that NH_4_^+^ was not detectable in the Lena transect (Table S1) and nitrate dropped down from 1.5 µmol L^−1^ to close to the detection limit during the first stations in the inner Delta (Fig. 4C). We speculate that phytoplankton, likely diatoms, are responsible for this uptake (Hawkings et al. 2017). This is backed up by the low silicate concentration in summer (Fig. 5), we found up to 60 µmol L^−1^ in summer and more than 100 µmol L^−1^ in winter. The SPM values, with high N percentage and low C/N ratios, further support this, as they indicate the production of fresh biomass (Supplementary Table S1).

We do not have direct evidence for DON uptake in summer, but according to Redfield ratios, silicate uptake must be backed up by approximately equimolar N uptake. N is limited in the delta, and must either stem from rapid recycling or from direct DON uptake as is known from permafrost-affected soils (Schimel and Bennett 2004) and also in aquatic environments (Wheeler et al. 1974).

Overall, we conclude that soils and pore water can be sources of NO_3_^−^ to the Lena Delta in winter, even though in low concentration (Heikoop et al. 2015) and that nitrate can also stem from in-situ nitrification. In summer, DIN overall is less important in the Delta, because it is used immediately, and we hypothesize that active DON uptake supports primary production in the water column of the Lena.

### Implications of thaw for the ecosystem

The flux of C and N from the Lena River has been estimated previously: Hugelius et al. (2020) estimated losses into aquatic systems (dissolved and particulate organic matter) to be 0.022 ± 0.02 Pg C·year^−1^ and 0.7 ± 0.5 Tg N·year^−1^. Wild et al. (2019) estimated an OC flux of 0.9 Tg C year^−1^ coming from permafrost and peat deposits. Based on the TDN data from the ArticGRO Station, an annual flux of approx. 170 Gg year^−1^ were estimated by Holmes et al (2012) for the year 2008 to 2012. In our single-year study from September 2018 to September 2019, we estimate an annual TN flux of 242.1 Gg year^−1^. This is higher than the average of the ArcticGRO data.

Comparisons between years and between different locations along the Lena River are challenging due to the two factors as follows: (1) The differences that can be accounted to strongly varying seasonal differences and (2) geographical differences, e.g., sources of different regions of the catchment.

Additionally some issues have to be considered and discussed, for instance, the particulate nitrogen is missing in the ArcticGRO data. Based on data from the cruises in this study, approximately one-fifth of the TN is particulate N, (average TN 16.0 µmol L^−1^, average TDN 12.9 µmol L^−1^) so this can partly explain the higher annual fluxes, we found. However, if we added 20 percent of N to the ArcticGRO data, the results still fall below our estimate of annual N flux by about 15% (approx.. 36 Gg), even though the N-NO_3_^−^ load is comparable (24.2 Gg in our study compared to 24.0 Gg, Holmes et al. 2012). It is possible that this deviation merely represents inter-annual variability because we compare one single annual cycle to a long-term data set. However, our Samoylov Island monitoring data were collected at higher temporal resolution and closer to the mouth of the Lena than the ArcticGRO data. In particular, the higher temporal resolution improved flux estimates by capturing the higher nutrient concentrations during peak discharge in spring and short hydrological events.

Additionally, 2019, our year of study, was a dry year and the summer discharge was low. There was no correlation of TN concentration with discharge (Fig. 6). Especially during the ice-covered period, higher concentrations of TN were measured during relatively low discharge. During winter, TN values varied strongly and ranged between 20 and 90 µmol L^−1^. Under ice, the higher velocity and pressure can re-suspend materials from the sediment. The TN concentrations during the summer cruise in the delta were slightly higher than our annual station data, which could be an evidence that the delta itself adds a considerable N load to the Lena River (Fig. 6). Fuchs et al. (2020) calculated an annual eroded N amount to the Lena River from the Sobe-Sise Cliff (Fig. 1C) of 0.4 × 10^6^ kg in the period of 2015–2018. The island is located downstream from Samoylov Island. Our soil incubations highlight that intense input can occur upon remineralization. Especially soils that were recently exposed by erosion are organic-rich and can release high amounts of DIN and DON.

This eroded soil material partly settled in the delta, in regions with low flow velocities, like on the beach of Samoylov Island. In the main channel, flow velocity is too high to allow for settling of organic-rich SPM. Instead, it is transported to the Laptev Sea, where it can be remineralized. However, only a minor part of the sediments transported by the Lena River enters the Laptev Sea shelf through the main channels of the delta, while the rest is dispersed within the network of the Lena Delta (Rachold et al. 1996).

Thibodeau et al. (2017) showed that the Lena River discharge mostly affects Laptev Sea surface water, while the bottom water is influenced by modified Atlantic‐derived water flowing along the continental slope and branching onto the shelf. Consequently, most ecosystem implications of nutrient supply from the Lena River affect the upper shelf waters. Secondary advective processes by currents can be responsible for along-shore drift of suspended OM once it discharges into the shelf area. Although our study and other previously published work identify high N:P export from the Lena Delta, these high N:P ratios are quickly lost and replaced with lower marine N:P ratios (Tuerena et al. 2022). This suggests biological uptake and sedimentary denitrification that lead to a loss of N on the Laptev Sea shelf.

### Potential N_2_O production in deposited and former eroded material

To investigate the potential N_2_O production and further emission to the atmosphere, we incubated samples from different soil types in Lena water over 8 weeks. In most of our incubations, the N_2_O concentrations were close to the detection limit but one incubation was a significant exception. From the sample of an organic-rich buried layer on the beach of the floodplain of Samoylov Island, significant amounts of N_2_O were produced with approximately 36 pg N-N_2_O g dw^−1^ day^−1^. Taking soil density into account, this corresponds to approximately 1000 μg N_2_O-N m^−2^ day^−1^_._ In this calculation, we ignored potential consumption of N_2_O. Actual emissions require chamber measurements, and we acknowledge that this single measurements may estimate potential N_2_O emission. However, higher N_2_O production rates of 1080 to 10,030 μg N_2_O-N m^−2^ day^−1^ were determined in unvegetated permafrost peat soils (Marushchak et al. 2011). Voigt et al (2020) summarized that unvegetated soils generally show highest N_2_O. Assuming that bare soils contribute to N_2_O emissions, the unvegetated floodplain in the delta, which contains layers of organic-rich deposits is a potential source of N_2_O emissions. Higher erosion rates, organic matter depositions within the delta, and higher NH_4_^+^ and NO_3_^−^ load in the water column can trigger the N_2_O emissions in the future.

## Conclusions

We found the Lena Delta region to play an important role as a source of reactive nitrogen stemming from the soil active layer and from thawing permafrost. Eroded soil materials release reactive, inorganic nitrogen and show significant rates or remineralization and nitrification. The inorganic nitrogen is almost completely utilized in the summer month and output to the coastal ocean is mainly organic nitrogen. Our estimate of nitrogen inputs in 2019 was 15% above the average data from the ArcticGRO stations further upstream in the Lena River. Though based on one annual cycle, this points towards additional N-sources downstream of the ArcticGRO station. We attribute this difference to an underestimation of the particulate nitrogen flux from eroded soil material in summer and due to resuspension of sediments, particularly important in winter.

Despite these high N fluxes, the effect of N on primary production on the shelf to date appears limited. However, the erosion and especially input of organic-rich material, and its subsequent remineralization from organic matter over DON to DIN, potentially may increase the N_2_O production and emissions from permafrost-affected soils and in permafrost-influenced aquatic environments, though this must be affirmed by future measurements.

Potentially, the changes in release of organic and inorganic nitrogen from thawing permafrost might alleviate the nitrogen limitations, in soils, the river, and consequently also in the coastal water of the Arctic Ocean causing higher productivity at the primary producer level and across food webs.

## Societal and policy implications

The Arctic is heavily impacted by climate change. Its air temperature increase is twice that of temperate regions (Smith et al. 2019). This increase in temperature is tied to extended thaw of permafrost-affected soils and increased soil organic matter decomposition. This increases the ubiquitous input of nutrients to the coastal ecosystem and enhances biogeochemical processes and availability of reactive nitrogen in the river and adjacent ocean. On a global scale, anthropogenic input of reactive nitrogen to the natural nitrogen cycle mainly by fertilization and industrial emission had imbalanced the cycle and these already exceed planetary boundaries (Steffen et al. 2011). That does not apply only to the over-fertilized temperate regions, but also to more pristine and oligotrophic ecosystems like the Arctic. Nonetheless, new data (Tuerena et al. 2022) suggest that the effect may be limited to the shelf region due to limited export to the open Arctic Ocean. Changing nutrient inputs potential increases coastal productivity, but temperature changes will also change the species composition in the coastal region. With possible increase in productivity, the ecosystem will be exposed to the increase in anthropogenic pressure, likely from fisheries.

The permafrost region is indicated as one tipping point regarding green-house gas emission of carbon dioxide and methane for the global climate system (Lenton et al. 2019). The main focus is usually put on methane and CO_2_ emissions, but it is yet unclear whether nitrous oxide production in Arctic soils and possibly in the rivers may intensify in the future and maybe contributed in particular to the tipping point. Global N_2_O emissions are increasing. Although atmospheric N_2_O is less concentrated than CO_2_ and CH_4_, it traps around 300 times more heat as a green-house gas. The N_2_O emissions from Arctic ecosystems could be one missing source in the global estimation.

The changing natural N cycle in the Arctic and the anthropogenic changed N cycle have to be considered together in the future.

## Supplementary Information

Below is the link to the electronic supplementary material.

Supplementary file 1 (PDF 2051 kb)
