# Supplementary material for: Seasonal nitrogen fluxes of the Lena River Delta
Source: Ambio. 2021 Dec 16;51(2):423–38. doi: 10.1007/s13280-021-01665-0 (PMC8692507; doi:10.1007/s13280-021-01665-0)
Supplement: Supplementary file 1 — (PDF 2051 kb) [file 13280_2021_1665_MOESM1_ESM.pdf]

Ambio

Electronic Supplementary Material

This supplementary material has not been peer reviewed.

Title: **Seasonal Nitrogen Fluxes of the Lena River Delta**

Authors: *Tina Sanders, Claudia Fiencke, Matthias Fuchs, Charlotte Haugk, Bennet Juhles, Gesine Mollenhauer, Olga Ogneva, Juri Palmtag, Vasily Povazhniy, Jens Strauss, Robyn Tuerena, Nadine Zell, and Kirstin Dähnke*

S 1: Supplementing information

Soil analysis regarding total carbon and nitrogen content

In soil and sediment samples total soil carbon and nitrogen were measured with an elemental analyzer (VarioMAX Elementar Analysensysteme GmbH, Hanau, Germany) after the soil has been sieved (< 2 mm), milled, and dried at 105 °C.

Soil Texture analysis

For texture analysis, the samples were digested with H_2O_2 to destroy organic matter when TOC content was >1 % and with HCl to destroy carbonates when $CaCO_3$ content was >2 %. The sand fractions were determined by dry sieving at four mesh sizes (630, 200, 125,63 µm). Silt fractions and clay were measured according to DIN ISO 11277 (Sedimat 4–12, UGT GmbH, Müncheberg,Germany).

KCl extraction for dissolved inorganic nitrogen (DIN) in soils

Inorganic nitrogen compounds were extracted from 10 g moist soil with 20 ml of 0.0125 mM KCl solution. For determination of nitrate, nitrate and ammonium concentrations, frozen KCl extracts were transported to Hamburg, where nitrate (and nitrite) concentrations were measured by HPLC (MEINKE et al. 1992). In all samples, nitrite was not detectable (below1µnol L^{-1}).Ammonium concentrations by photometer (lowest detectable value 1 µmol l^{-1}) (DIN 38406–E5-1).

Suspended particulate matter (SPM)

Filter samples, were dried at 50 °C and weighed for later determination of C/N ratios, suspended particulate matter (SPM) content, and δ15N-SPM analysis. C/N ratios were determined with an elemental analyzer (Eurovector EA 3000) calibrated against a certified acetanilide standard (IVA Analysentechnik, Germany). The standard deviation of C/N analysis was 0.05 % for carbon and 0.005 % for nitrogen.

$\delta^{15}N$ SPM was analysed with an element analyser (Carlo Erba NA 2500) coupled with an isotope ratio mass spectrometer (Finnigan MAT 252).) where the standards for nitrogen and oxygen are atmospheric N_2 and Vienna Standard Mean Ocean Water (VSMOW), respectively.

International isotope standards IAEA N1, IAEA N2, and a certified sediment standard (IVA Analysentechnik, Germany) for suspended matter isotope values.

Dissolved Organic carbon (DOC)

Water samples for DOC analysis were filtered through 0.7 µm pore-size filters in the field, acidified with 20 µl concentrated (35%) hydrochloric acid (HCl) and kept cool in a lab refrigerator at 4°C. DOC was measured as non–purgeable organic carbon (NPOC) using a total organic carbon analyser TOC-VCPH/CPN from SHIMADZU at AWI Potsdam. Two mL of water were diluted with 10 mL of distilled water and filled into small test tubes. The tubes were placed into an auto-sampler. During the measurement, sparge gas was first bubbled through the sample and hydrochloric acid was added to remove inorganic carbon by converting it to carbon dioxide. The remaining organic carbon is measured by combusting the sample at 680°C and measuring the CO_2 concentration. To ensure the accuracy of the measurement, blank samples of ultra-pure water and standards with known DOC concentrations were run before and after the measurement and then in regular intervals.

Total and dissolved organic Nitrogen (TN/TDN) and Phosphorus (TP/TDP)

For the determination of DON, we used the persulfate oxidation method (Knapp et al., 2005). The first step is the oxidation of total dissolved nitrogen (TDN, the sum of nitrate, nitrite, ammonium and DON) to nitrate. For the oxidation of TDN to nitrate, 24 ml of the sample plus 2 ml of persulfate oxidizing reagent (POR) was added to a Teflon bottle. The POR contains ACS-grade sodium hydroxide, certified ACS-grade boric acid and certified ACS-grade potassium persulfate, which was recrystallized three times (Hansen and Koroleff 2007). For the digestion a microwave (CEM, Mars 5) was used. The reagent blank was always <2 µM. DON concentrations were calculated by subtracting the DIN concentrations. As reference, internal standards of ammonium sulfate and urea were used.

In the same digestion also the total phosphorus were measured. Reagent blank was below 0,1 µM.

For the analysis of total nitrogen and phosphorus unfiltered we have used the same method using unfiltered water samples.

Inorganic nutrient analysis

All filtered water samples were analyzed in duplicate for concentration of ammonium, nitrite, nitrate, phosphate and silicate using an automated continuous flow system (AA3, Seal

Analytical, Germany) and standard colorimetric techniques (Hansen and Koroleff 2007). Detection limits were 1 μmol L^{-1} for nitrate and silicate, 0.5 μmol L^{-1} for nitrite, ammonium and phosphate.

Nutrient Analysis of Lena River Monitoring Program by the OSL

Inorganic nutrients (nitrate, nitrite, ammonium, silicate and phosphate), TN samples from Lena River Monitoring program were delivered from sampling site and stored frozen (-24°C) until analyzed on automated continuous flow system (San+, SKALAR, Netherlands) with standard colorimetric techniques (Aminot et al. 2009) . TN protocols were identical to above mentioned technique of peroxide-based wet oxidation with microwave digestion replaced by autoclaving at 121°C. Environmental matrix reference materials (Environment and Climate Change Canada) were used as tracking standard in every batch of samples.

N_2O production measurements

Potential Aerobic nitrous oxide production rates were determined in incubation tests with the soil samples taken during expedition in August 2019, directly frozen in the field and thawed in a refrigerator for three days in March 2019. After removing stones, roots and other plant material samples were homogenized by sieving for mineral soils (2 mm mesh size) or by hand mixing and cutting with scissors for peat rich material. 1 g fresh weight of homogenized soil were weighted in 100-ml-serum bottles, 20 ml of Lena water were added and and the bottle were sealed air-tight with rubber thick septa. Soil samples were incubated at 5 °C, without shaking in the dark in 18 replicates. At the begining, and after 1, 2, 8, 11, 18 weeks 1ml-gas samples were taken from three samples and concentrations of N_2O was determined by gas chromatography (GC, Agilent Technologies 7890 A, Santa Clara, CA, USA). Before the gas measurements at GC, partial pressure were measured in all flasks (KELLER, Mano 2000 Leo 1, Switzerland). In case of low pressure in incubation flasks, 500 μl N_2 was injected. The injection volume for gas analysis was 250 μL. Gases were separated on a Porapak Q column (1.8 m length, 2 mm ID) and quantified with an electron capture detector (ECD). The inflow, oven, and detector temperature was 360 °C (ECD), respectively. Nitrogen served as the carrier gas (30 mL min^{-1}). The mixing ratios (ppm) were calculated from the peak areas based on a standard curve with 3 different levels of N_2O concentrations, ranging from 0.29-1.55 ppm. The total amount of N_2O was calculated from the partial pressure of the gases, the

temperature, the headspace volume, the amount of water and the water solubility of N_2O. Aerobic N_2O production only started after a lag phase, so production rates were calculated as a function of time after 8 weeks of incubation, where maximal N_2O was produced over time.

Supplementary Table S1
Data are related to https://doi.pangaea.de/10.1594/PANGAEA.933187

Sample Number	Longitude	Latitude	Date	Bot. Depth	Temperature	Salinity
**SUMMER**				m	°C	PSU
LEN19_S_01_01	126.695646	72.3993839	2019-08-09	19	15.95	0.08
LEN19_S_01_18	126.695646	72.3993839	2019-08-09	19	15.99	0.08
LEN19_S_02_1	126.9289955	72.5373429	2019-08-09	17	15.95	0.08
LEN19_S_02_16	126.9289955	72.5373429	2019-08-09	17	15.97	0.08
LEN19_S_02_Bottom	126.9289955	72.5373429	2019-08-09	17	15.98	0.08
LEN19_S_03_1	127.4193214	72.6270543	2019-08-09	6	15.74	0.08
LEN19_S_04_1	127.9591821	72.6335186	2019-08-09	3	15.18	0.08
LEN19_S_05_1	128.2446629	72.5638046	2019-08-09	5	15.25	0.08
LEN19_S_05_4	128.2446629	72.5638046	2019-08-09	5	15.22	0.08
LEN19_S_06_1	128.5154812	72.5211118	2019-08-08	8	15.49	0.08
LEN19_S_06_6	128.5154812	72.5211118	2019-08-08	8	15.49	0.08
LEN19_S_07_1	128.6950478	72.4613355	2019-08-08	18	14.99	0.08
LEN19_S_07_15	128.6950478	72.4613355	2019-08-08	18	15.02	0.08
LEN19_S_78_1	128.8410507	72.4530305	2019-08-08	10	15.00	0.08
LEN19_S_78_8	128.8410507	72.4530305	2019-08-08	10	15.01	0.08
LEN19_S_08_1	128.9707677	72.4770465	2019-08-08	8	14.93	0.08
LEN19_S_08_6	128.9707677	72.4770465	2019-08-08	8	14.91	0.08
LEN19_S_89_1	129.0992231	72.5016874	2019-08-08	13	14.98	0.08
LEN19_S_89_6	129.0992231	72.5016874	2019-08-08	13	14.75	0.08
LEN19_S_89_12	129.0992231	72.5016874	2019-08-08	13	14.56	0.08
LEN19_S_09_1	129.2484149	72.5090431	2019-08-08	11	14.91	0.08
LEN19_S_09_5	129.2484149	72.5090431	2019-08-08	11	14.45	0.08
LEN19_S_09_10	129.2484149	72.5090431	2019-08-08	11	14.45	0.08

Sample Number	Nitrite	Silicate	Phosphate	Ammonium	Nitrate
**SUMMER**	µmol L-1	µmol L-1	µmol L-1	µmol L-1	µmol L-1
LEN19_S_01_01	0.10	48.66	0.07	0.74	1.44
LEN19_S_01_18	0.11	25.75	0.07	0.76	1.38
LEN19_S_02_1	0.12	22.89	0.06	0.78	1.04
LEN19_S_02_16	0.09	60.57	0.06	0.77	1.09
LEN19_S_02_Bottom	0.15	21.79	0.09	0.76	1.22
LEN19_S_03_1	0.11	18.78	0.05	0.79	0.65
LEN19_S_04_1	0.12	18.78	0.05	0.75	0.28
LEN19_S_05_1	0.10	16.31	0.04	0.66	0.25
LEN19_S_05_4	0.10	17.05	0.04	0.65	0.25
LEN19_S_06_1	0.07	27.20	0.04	0.55	0.26
LEN19_S_06_6	0.10	20.47	0.05	0.47	0.21
LEN19_S_07_1	0.10	25.29	0.04	0.27	0.16
LEN19_S_07_15	0.08	39.95	0.04	0.35	0.25
LEN19_S_78_1	0.09	30.75	0.04	0.22	0.16
LEN19_S_78_8	0.07	59.03	0.05	0.26	0.18
LEN19_S_08_1	0.10	21.57	0.03	0.17	0.12
LEN19_S_08_6	0.09	24.37	0.03	0.16	0.15
LEN19_S_89_1	0.09	29.64	0.04	0.15	0.20
LEN19_S_89_6	0.09	29.98	0.03	0.15	0.19
LEN19_S_89_12	0.09	30.48	0.04	0.15	0.19
LEN19_S_09_1	0.10	29.10	0.04	0.15	0.20
LEN19_S_09_5	0.09	29.51	0.04	0.22	0.27
LEN19_S_09_10	0.10	32.72	0.04	0.23	0.27

Sample Number	TDN	DON	TDP	TN	TP	DOC	DON
**SUMMER**	µmol L-1	µmol L-1	µmol L-1	µmol L-1	µmol L-1	mg L-1	mg L-1
LEN19_S_01_01	16.13	14.59	0.14	15.67	0.40	5.44	0.20
LEN19_S_01_18	12.62	11.13	0.10	15.81	0.36	5.88	0.16
LEN19_S_02_1	11.49	10.33	0.09	14.68	0.32	5.53	0.14
LEN19_S_02_16	12.20	11.01	0.09	15.62	0.31	5.75	0.15
LEN19_S_02_Bottom	13.67	12.31	0.15	17.65	0.65		
LEN19_S_03_1	11.67	10.92	0.10	14.81	0.31	5.72	0.15
LEN19_S_04_1	12.32	11.92	0.10	15.09	0.32	5.68	0.17
LEN19_S_05_1	11.54	11.19	0.10	15.03	0.30	5.36	0.16
LEN19_S_05_4	12.20	11.86	0.12	17.21	0.45	5.54	0.17
LEN19_S_06_1	11.73	11.41	0.10	17.52	0.44	5.37	0.16
LEN19_S_06_6	11.92	11.62	0.08	17.56	0.47	5.92	0.16
LEN19_S_07_1	12.24	11.98	0.10	15.28	0.38	5.59	0.17
LEN19_S_07_15	12.94	12.60	0.10	15.80	0.36	5.36	0.18
LEN19_S_78_1	13.13	12.89	0.08	16.87	0.39	5.44	0.18
LEN19_S_78_8	13.58	13.33	0.09	15.48	0.38	5.72	0.19
LEN19_S_08_1	12.51	12.29	0.09	15.94	0.42	5.46	0.17
LEN19_S_08_6	12.72	12.48	0.10	15.02	0.40	5.49	0.17
LEN19_S_89_1	12.18	11.89	0.07	15.54	0.37	5.30	0.17
LEN19_S_89_6	12.90	12.62	0.09	16.51	0.37	5.28	0.18
LEN19_S_89_12	13.23	12.95	0.08	15.79	0.36	5.33	0.18
LEN19_S_09_1	13.26	12.95	0.07	15.66	0.35	5.55	0.18
LEN19_S_09_5	13.13	12.78	0.08	16.87	0.39	5.67	0.18
LEN19_S_09_10	12.31	11.94	0.08	15.63	0.36	5.53	0.17

Sample Number	SPM	Total Nitrogen	Total Carbon	C/N
**SUMMER**	mg L-1	[%]	[%]	
LEN19_S_01_01	12.54	0.55	4.49	8.74
LEN19_S_01_18	23.51	0.45	4.39	9.86
LEN19_S_02_1	9.52	0.55	4.33	7.89
LEN19_S_02_16	9.26	0.71	5.62	7.88
LEN19_S_02_Bottom				
LEN19_S_03_1	9.19	0.63	5.39	8.58
LEN19_S_04_1	9.31	0.66	5.47	8.29
LEN19_S_05_1	9.83	0.73	6.14	8.47
LEN19_S_05_4	11.26	0.69	5.95	8.64
LEN19_S_06_1	11.72	0.62	5.48	8.84
LEN19_S_06_6	11.40	0.70	6.05	8.59
LEN19_S_07_1	7.59	0.81	7.39	9.12
LEN19_S_07_15	9.46	0.78	6.43	8.28
LEN19_S_78_1	9.06	0.61	5.30	8.72
LEN19_S_78_8	10.02	0.71	7.02	9.91
LEN19_S_08_1	9.65	0.84	7.16	8.50
LEN19_S_08_6	9.74	0.60	5.46	9.13
LEN19_S_89_1	7.18	0.99	7.90	7.95
LEN19_S_89_6	7.69	1.09	8.57	7.88
LEN19_S_89_12	8.35	0.92	7.60	8.29
LEN19_S_09_1	5.92	1.37	9.93	7.22
LEN19_S_09_5	7.40	1.05	8.46	8.08
LEN19_S_09_10	7.22	1.22	8.87	7.29

Sample Number	TDN/TDP	DON/TDP	Nitrate / Phosphate	TN/TP	DOC/DON
**SUMMER**					
LEN19_S_01_01	112.80	102.01	21.82	38.88	26.65
LEN19_S_01_18	126.59	111.63	21.22	43.71	37.74
LEN19_S_02_1	132.60	119.24	18.16	46.41	38.2
LEN19_S_02_16	130.91	118.20	17.87	51.11	37.31
LEN19_S_02_Bottom	92.81	83.55	13.84	27.25	
LEN19_S_03_1	117.13	109.55	12.42	47.14	37.4
LEN19_S_04_1	123.59	119.57	5.62	47.71	34.05
LEN19_S_05_1	118.36	114.75	6.00	50.62	34.21
LEN19_S_05_4	104.30	101.34	5.68	38.57	33.38
LEN19_S_06_1	115.17	112.00	6.12	39.44	33.66
LEN19_S_06_6	144.74	141.08	4.58	37.69	36.39
LEN19_S_07_1	125.53	122.89	4.47	40.06	33.3
LEN19_S_07_15	132.67	129.25	6.02	43.92	30.35
LEN19_S_78_1	159.53	156.51	4.30	42.77	30.15
LEN19_S_78_8	149.24	146.48	3.93	41.06	30.62
LEN19_S_08_1	137.50	135.03	3.81	38.31	31.76
LEN19_S_08_6	133.43	130.95	4.35	37.87	31.41
LEN19_S_89_1	165.35	161.43	5.50	42.44	31.81
LEN19_S_89_6	138.49	135.47	5.70	44.29	29.88
LEN19_S_89_12	160.63	157.23	5.34	43.63	29.40
LEN19_S_09_1	179.97	175.83	5.37	44.35	30.59
LEN19_S_09_5	159.53	155.17	7.19	42.77	31.72
LEN19_S_09_10	145.69	141.33	7.13	42.94	33.06

Sample Number	Longitude	Latitude	Date	Bot. Depth	Temperature	Salinity
**SUMMER**				m	°C	PSU
CAC_04_1	130.1263035	72.5301281	2019-08-03	7	10.05	2.95
CAC_04_6	130.1263035	72.5301281	2019-08-03	7	4.56	18.78
CAC_04_Bottom	130.1263035	72.5301281	2019-08-03	7	3.87	22.91
CAC_05_1	130.4335066	72.5398332	2019-08-03	13	9.82	3.46
CAC_05_6	130.4335066	72.5398332	2019-08-03	13	5.82	14.74
CAC_05_12	130.4335066	72.5398332	2019-08-03	13	-0.05	27.64
CAC_05_Bottom	130.4335066	72.5398332	2019-08-03	13	-0.03	27.67
CAC_06_1	130.7224841	72.5411834	2019-08-03	17	9.06	4.10
CAC_06_8	130.7224841	72.5411834	2019-08-03	17	5.54	15.26
CAC_06_15	130.7224841	72.5411834	2019-08-03	17	-0.60	27.89
CAC_06_Bottom	130.7224841	72.5411834	2019-08-03	17	-0.62	27.94
CAC_07_1	131.0183698	72.5505630	2019-08-03	20	9.07	4.87
CAC_07_8	131.0183698	72.5505630	2019-08-03	20	3.58	18.40
CAC_07_18	131.0183698	72.5505630	2019-08-03	20	-0.87	28.27
CAC_07_Bottom	131.0183698	72.5505630	2019-08-03	20	-0.90	28.29
CAC_08_1	131.3146861	72.5544617	2019-08-03	21	8.81	3.95
CAC_08_10	131.3146861	72.5544617	2019-08-03	21	-0.41	25.34
CAC_08_19	131.3146861	72.5544617	2019-08-03	21	-1.03	28.92
CAC_08_Bottom	131.3146861	72.5544617	2019-08-03	21	-0.97	28.94
CAC_09_1	131.6060619	72.5588626	2019-08-03	22	6.67	2.37
CAC_09_10	131.6060619	72.5588626	2019-08-03	22	1.00	20.44
CAC_09_20	131.6060619	72.5588626	2019-08-03	22	-1.10	29.33
CAC_09_Bottom	131.6060619	72.5588626	2019-08-03	22	-1.09	29.36
CAC_10_1	131.9148058	72.5532083	2019-08-03	22	5.55	2.70
CAC_10_10	131.9148058	72.5532083	2019-08-03	22	0.12	18.60
CAC_10_20	131.9148058	72.5532083	2019-08-03	22	-1.08	29.17
CAC_10_Bottom	131.9148058	72.5532083	2019-08-03	22	-1.04	29.14

Sample Number	Nitrite	Silicate	Phosphate	Ammonium	Nitrate
**SUMMER**	µmol L-1	µmol L-1	µmol L-1	µmol L-1	µmol L-1
CAC_04_1	0.05	14.62	0.04	0.14	0.05
CAC_04_6	0.11	28.08	0.31	0.86	1.83
CAC_04_Bottom	0.21	32.49	0.24	6.56	3.44
CAC_05_1	0.05	16.93	0.03	0.11	0.01
CAC_05_6	0.08	21.97	0.12	1.54	0.07
CAC_05_12	0.09	31.46	0.58	0.32	5.62
CAC_05_Bottom	0.08	33.67	0.67	0.47	5.58
CAC_06_1	0.11	17.73	0.03	0.61	0.04
CAC_06_8	0.06	29.75	0.11	0.23	0.13
CAC_06_15	0.07	32.45	0.23	0.23	1.66
CAC_06_Bottom	0.10	33.04	0.67	0.23	6.24
CAC_07_1	0.07	17.73	0.04	0.14	0.02
CAC_07_8	0.08	29.75	0.06	0.28	0.05
CAC_07_18	0.06	32.77	0.78	0.08	6.13
CAC_07_Bottom	0.10	41.74	1.12	0.72	6.40
CAC_08_1	0.14	19.93	0.10	0.16	0.00
CAC_08_10	0.09	36.38	0.18	0.15	2.45
CAC_08_19	0.05	30.56	0.81	0.08	5.65
CAC_08_Bottom	0.05	30.98	0.79	0.13	5.41
CAC_09_1	0.15	11.87	0.05	0.52	0.73
CAC_09_10	0.17	28.37	0.07	1.53	0.69
CAC_09_20	0.11	28.66	0.90	0.11	5.20
CAC_09_Bottom	0.05	28.43	0.81	0.15	5.18
CAC_10_1	0.23	14.17	0.03	0.44	0.07
CAC_10_10	0.19	35.22	0.16	0.56	2.03
CAC_10_20	0.06	30.20	0.80	0.31	5.50
CAC_10_Bottom	0.07	35.20	0.59	0.27	3.62

Sample Number	TDN	DON	TDP	TN	TP	DOC	DON
**SUMMER**	µmol L-1	µmol L-1	µmol L-1	µmol L-1	µmol L-1	mg L-1	mg L-1
CAC_04_1	13.48	13.39	0.10	14.44	0.31	7.03	0.19
CAC_04_6	14.36	12.43	0.35	21.48	1.52	4.76	0.17
CAC_04_Bottom	19.37	15.71	0.28	54.52	4.46		0.22
CAC_05_1	14.25	14.18	0.09	15.45	0.27	6.63	0.20
CAC_05_6	11.26	11.11	0.16	13.54	0.32	5.83	0.16
CAC_05_12	15.49	9.78	0.62	16.02	1.11	3.32	0.14
CAC_05_Bottom	16.27	16.27	0.71	35.91	3.08		0.15
CAC_06_1	13.91	13.76	0.14	14.85	0.28	8.14	0.19
CAC_06_8	14.46	14.27	0.18	12.05	0.32	6.38	0.20
CAC_06_15	14.73	13.00	0.32	15.41	0.56	3.14	0.18
CAC_06_Bottom	18.92	12.59	0.70	31.54	3.87		0.18
CAC_07_1	15.40	15.31	1.07	16.75	0.29	7.73	0.21
CAC_07_8	13.67	13.54	0.14	18.19	0.37	6.71	0.19
CAC_07_18	14.46	8.28	0.14	37.90	4.10	9.76	0.12
CAC_07_Bottom							
CAC_08_1	16.06	15.93	0.14	14.94	0.13	7.60	0.22
CAC_08_10	17.01	14.47	0.20	25.29	0.30	6.64	0.20
CAC_08_19	13.24	7.54	0.84	13.63	1.08	2.48	0.11
CAC_08_Bottom	14.45	8.99	0.82	31.42	4.01		0.13
CAC_09_1	24.02	23.14	0.19	17.89	0.47	8.65	0.32
CAC_09_10	15.84	14.98	0.15	17.95	0.28	6.59	0.21
CAC_09_20	12.18	6.87	0.81	12.95	0.87	2.23	0.10
CAC_09_Bottom	13.14	7.91	0.83	28.97	3.39		0.11
CAC_10_1	17.41	17.11	0.17	19.19	0.32	8.44	0.24
CAC_10_10	15.67	13.46	0.25	10.86	0.29	6.55	0.19
CAC_10_20	14.11	8.55	0.82	32.50	0.99	2.40	0.12
CAC_10_Bottom	14.94	11.25	0.60	68.50	3.88		0.16

Sample Number	SPM	Total Nitrogen	Total Carbon	C/N
**SUMMER**	mg L-1	[%]	[%]	
CAC_04_1	4.25	1.16	10.17	8.76
CAC_04_6	63.20	0.33	4.22	12.99
CAC_04_Bottom				
CAC_05_1	2.78	1.35	11.45	8.48
CAC_05_6	3.74	0.95	8.13	8.53
CAC_05_12	14.80	0.33	4.19	12.68
CAC_05_Bottom				
CAC_06_1	1.85	2.09	17.81	8.53
CAC_06_8	3.04	1.08	8.82	8.18
CAC_06_15	7.17	0.31	3.88	12.39
CAC_06_Bottom				
CAC_07_1	1.40	2.89	23.99	8.31
CAC_07_8	2.00	2.19	21.48	9.79
CAC_07_18	11.29	0.18	2.45	13.33
CAC_07_Bottom				
CAC_08_1	2.05	2.45	18.60	7.59
CAC_08_10	2.32	1.25	10.86	8.70
CAC_08_19	12.09	0.21	3.25	15.25
CAC_08_Bottom				
CAC_09_1	1.42	3.12	21.99	7.05
CAC_09_10	2.47	1.11	9.14	8.27
CAC_09_20	5.84	0.33	4.00	12.16
CAC_09_Bottom				
CAC_10_1	1.78	2.87	21.33	7.44
CAC_10_10	2.67	1.45	11.66	8.03
CAC_10_20	9.21	0.25	2.67	10.73
CAC_10_Bottom				

Sample Number	TDN/TDP	DON/TDP	Nitrate / Phosphate	TN/TP	DOC/DON
**SUMMER**					
CAC_04_1	129.65	128.74	1.24	47.28	37.57
CAC_04_6	40.66	35.19	5.95	14.16	27.38
CAC_04_Bottom	68.24	55.37	14.53	11.97	
CAC_05_1	152.95	152.23	0.46	57.06	33.4
CAC_05_6	69.31	68.38	0.56	42.24	37.44
CAC_05_12	25.08	15.83	9.64	14.47	24.22
CAC_05_Bottom	22.89	14.93	8.35	11.66	
CAC_06_1	100.30	99.22	1.13	53.77	42.26
CAC_06_8	79.44	78.40	1.12	37.98	31.91
CAC_06_15	46.24	40.82	7.24	27.30	18.71
CAC_06_Bottom	26.96	17.94	9.25	8.14	
CAC_07_1	14.36	14.27	0.42	56.83	36.08
CAC_07_8	100.13	99.20	0.82	49.39	35.38
CAC_07_18	102.71	58.81	7.86	9.,25	84.19
CAC_07_Bottom					
CAC_08_1	115.63	114.88	0.00	114.92	34.07
CAC_08_10	87.21	74.19	13.86	83.97	32.79
CAC_08_19	15.83	9.02	6.98	12.66	23.48
CAC_08_Bottom	17.64	10.97	6.89	7.83	
CAC_09_1	124.57	119.99	14.62	38.23	26.72
CAC_09_10	107.49	101.65	10.31	63.24	31.42
CAC_09_20	44270	8.48	5.76	14.84	24.47
CAC_09_Bottom	15.84	9.53	6.41	8.55	
CAC_10_1	100.46	98.71	2.28	60.66	35.21
CAC_10_10	62.90	54.01	12.90	37.68	34.76
CAC_10_20	17.27	10.47	6.87	32.75	20.07
CAC_10_Bottom	24.80	18.68	6.18	17.65	

Sample Number	Longitude	Latitude	Date	Bot. Depth	Temperature	Salinity
**WINTER**				m	°C	PSU
CAC19_01_2.5	129.2480173	72.5090386	2019-03-29	11	0.08	0.21
CAC19_01_6	129.2480173	72.5090386	2019-03-29	11	0.09	0.21
CAC19_01_11	129.2480173	72.5090386	2019-03-29	11	0.05	0.21
CAC19_02_2.5	129.5455126	72.5168283	2019-03-29	3	0.06	0.20
CAC19_03_3	129.8419953	72.5253607	2019-03-30	4	0.05	0.21
CAC19_04_3	129.8638871	72.5254943	2019-03-31	3	0.10	0.21
CAC19_23_2	129.6930193	72.5213603	2019-03-31	3	0.13	0.21
CAC19_A_2	129.1016438	72.5012794	2019-04-01	12	0.10	0.21
CAC19_A_7	129.1016438	72.5012794	2019-04-01	12	0.06	0.21
CAC19_A_11	129.1016438	72.5012794	2019-04-01	12	0.05	0.21
CAC19_B_2	128.9711000	72.4793812	2019-04-01	6	0.11	0.21
CAC19_B_5	128.9711000	72.4793812	2019-04-01	6	0.02	0.21
CAC19_C_2	128.8445192	72.455611	2019-04-02	2	0.03	0.21
CAC19_D_2	128.6944962	72.4615553	2019-04-02	19	0.05	0.21
CAC19_D_10	128.6944962	72.4615553	2019-04-02	19	0.05	0.20
CAC19_D_18	128.6944962	72.4615553	2019-04-02	19	0.06	0.21
CAC19_E_2.5	128.6297501	72.5019032	2019-04-03	3	0.13	0.19
CAC19_F_3	128.4921886	72.5187824	2019-04-03	4	0.11	0.25
CAC19_G_2.5	128.3532643	72.535431	2019-04-04	9	0.18	0.21
CAC19_G_7.5	128.3532643	72.535431	2019-04-04	9	0.12	0.21
CAC19_H_3	128.2384589	72.5640799	2019-04-04	4	0.11	0.21

**Sample Number**	**Nitrite**	**Silicate**	**Phosphate**	**Ammonium**	**Nitrate**
**WINTER**	µmol L-1	µmol L-1	µmol L-1	µmol L-1	µmol L-1
CAC19_01_2.5	0.15	100.93	0.10	0.89	12.58
CAC19_01_6	0.11	112.99	0.10	0.98	12.68
CAC19_01_11	0.12	109.18	0.13	1.63	12.30
CAC19_02_2.5	0.09	120.26	0.11	0.89	9.59
CAC19_03_3	0.13	82.94	0.08	0.95	11.25
CAC19_04_3	0.16	118.32	0.10	0.82	10.70
CAC19_23_2	0.18	111.85	0.10	0.77	10.39
CAC19_A_2	0.10	104.95	0.11	1.01	9.84
CAC19_A_7	0.10	107.54	0.11	0.90	10.37
CAC19_A_11	0.08	104.46	0.09	0.95	10.64
CAC19_B_2	0.15	99.13	0.09	1.04	10.17
CAC19_B_5	0.09	107.29	0.09	0.91	10.46
CAC19_C_2	0.13	115.05	0.10	0.96	10.01
CAC19_D_2	0.13	111.24	0.10	0.95	8.83
CAC19_D_10	0.09	115.16	0.10	0.82	8.69
CAC19_D_18	0.12	103.12	0.08	0.91	10.25
CAC19_E_2.5	0.11	95.32	0.09	0.80	8.79
CAC19_F_3	0.30	142.42	0.12	14.63	7.79
CAC19_G_2.5	0.12	91.47	0.09	1.52	9.87
CAC19_G_7.5	0.12	104.51	0.09	0.96	8.97
CAC19_H_3	0.08	119.38	0.10	0.91	7.59

**Sample Number**	**TDN**	**DON**	**TDP**	**DOC**	**DON**
**WINTER**	µmol L-1	µmol L-1	µmol L-1	mg L-1	mg L-1
CAC19_01_2.5	24.19	10.58	0.16	6.09	0.15
CAC19_01_6	24.15	10.38	0.15	6.13	0.15
CAC19_01_11	27.95	13.90	0.20	6.28	0.19
CAC19_02_2.5	21.21	10.65	0.17	7.30	0.15
CAC19_03_3	25.12	12.78	0.14	6.02	0.18
CAC19_04_3	23.53	11.85	0.16	6.61	0.17
CAC19_23_2	22.86	11.52	0.13	6.11	0.16
CAC19_A_2	23.95	13.01	0.16	6.01	0.18
CAC19_A_7	22.58	11.21	0.15	6.17	0.16
CAC19_A_11	24.38	12.71	0.15	5.94	0.18
CAC19_B_2	23.70	12.34	0.23	6.12	0.17
CAC19_B_5	24.60	13.14	0.15	5.91	0.18
CAC19_C_2	24.49	13.39	0.15	6.13	0.19
CAC19_D_2	22.86	12.95	0.16	6.55	0.18
CAC19_D_10	20.15	10.55	0.14	5.97	0.15
CAC19_D_18	22.60	11.32	0.15	6.72	0.16
CAC19_E_2.5	21.19	11.50	0.14	5.82	0.16
CAC19_F_3	34.39	11.67	0.22	8.54	0.16
CAC19_G_2.5	23.58	12.08	0.15	6.05	0.17
CAC19_G_7.5	20.83	10.80	0.17	6.12	0.15
CAC19_H_3	21.20	12.62	0.14	6.02	0.18

Sample Number	TDN/TDP	DON/TDP	Nitrate / Phosphate	DOC/DON
**WINTER**				
CAC19_01_2.5	147.40	64.48	122.73	41.12
CAC19_01_6	162.12	69.70	129.36	42.15
CAC19_01_11	139.10	69.19	96.09	32.28
CAC19_02_2.5	121.25	60.85	86.40	48.99
CAC19_03_3	173.67	88.39	149.03	33.65
CAC19_04_3	151.33	76.21	102.93	39.85
CAC19_23_2	173.66	87.52	104.44	37.91
CAC19_A_2	145.95	79.28	93.67	33.10
CAC19_A_7	151.56	75.24	96.49	39.32
CAC19_A_11	166.11	86.62	116.30	33.39
CAC19_B_2	101.52	52.84	114.28	35.44
CAC19_B_5	162.79	86.97	115.56	32.11
CAC19_C_2	166.86	91.23	98.17	32.67
CAC19_D_2	141.15	79.95	84.92	36.13
CAC19_D_10	139.32	72.92	86.93	40.41
CAC19_D_18	151.72	75.99	135.76	42.39
CAC19_E_2.5	153.41	83.23	97.66	36.19
CAC19_F_3	157.56	53.47	64.66	52.29
CAC19_G_2.5	158.32	81.08	111.56	35.76
CAC19_G_7.5	119.08	61.71	104.87	40.47
CAC19_H_3	151.15	89.97	74.09	34.05
